# Association between plasma angiotensin converting enzyme 1 level and cognition over 12‐years: The Look AHEAD Study

**DOI:** 10.1002/alz.089526

**Published:** 2025-01-09

**Authors:** Sevil Yasar, Andrea Anderson, Kathleen M. Hayden, Mark A. Espeland, Owen T. Carmichael, Jeanne M Clark, Michelle C Carlson, Daniel Asby, Patrick Gavin Kehoe, James Scott Miners

**Affiliations:** ^1^ Johns Hopkins University School of Medicine, Baltimore, MD USA; ^2^ Wake Forest School of Medicine, Winston‐Salem, NC USA; ^3^ Wake Forest University School of Medicine, Winston Salem, NC USA; ^4^ Pennington Biomedical Research Center, Baton Rouge, LA USA; ^5^ Johns Hopkins School of Medicine, Baltimore, MD USA; ^6^ Johns Hopkins Bloomberg School of Public Health, Baltimore, MD USA; ^7^ University of Bristol, Medical School, Bristol UK; ^8^ University of Bristol, Bristol UK; ^9^ University of Bristol, Bristol, Horfield UK

## Abstract

**Background:**

The renin angiotensin system (RAS) has been proposed as a potential modifier of the development of Alzheimer’s disease (AD). However, prospective studies of RAS are sparse especially among cognitively normal individuals with type 2 diabetes mellitus (T2DM) and other vascular risk factors. We aimed to determine whether plasma levels or activity of the RAS marker ACE‐1 predicts cognitive decline over an 8‐year period in this population.

**Method:**

We performed a secondary data analysis of the Action for Health in Diabetes (Look AHEAD) study among community‐dwelling, non‐demented adults with overweight/obesity and T2DM aged 45‐76 years at baseline observed over a 12‐year period. Of 5,145 participants, we included 310 participants who were not using medications affecting the RAS system, had blood available at years 1, 4 and 10 and had undergone cognitive testing at least once and up to 4 times between years 8 and 16. Plasma ACE‐1 was measured using by ELISA (R&D systems) and ACE‐1 activity using immunocapture fluorogenic peptide assay. Cognitive tests included the Modified Mini‐Mental State Examination (3MSE), the Trail Making Test Parts A and B, the Stroop Color‐Word Test, the Digit Symbol Substitution Test, and the Rey Auditory Verbal Learning (RAVLT) Immediate and Delayed test. A cognitive composite score was derived by averaging standardized cognitive test scores. Mixed effects models were used to relate log converted ACE‐1 levels and activity to performance in cognitive test scores after adjusting for potential confounders.

**Result:**

Participants included in the analyses were more likely to be female (65%), White (76%), and were more likely to have obesity (34%), have lower HgbA1C, LDL and systolic blood pressure control compared to the excluded particpants. Higher mean ACE‐1 levels measured at Y1, 4 and 10 was significantly associated with 3MSE (β=0.1774; p=0.02) and RAVLT‐delayed recall (β=0.1600; p=0.05). The cognitive composite score was not associated with ACE‐1 level. There was no association between ACE‐1 activity and cognitive function.

**Conclusion:**

In this sample with T2DM and dementia free at baseline, we observed longitudinal associations between mean ACE‐1 levels and global cognition and memory.

